# Evaluating sensitivity and specificity of the DPP Vet TB assay in badgers using Bayesian latent class models

**DOI:** 10.1371/journal.pone.0313825

**Published:** 2025-03-04

**Authors:** Rachel C. Jinks, Sandrine Lesellier, Freya Smith, Fraser D. Menzies, Roland T. Ashford, Laura Waring, Dipesh Dave, Paul Anderson, Lesley A. Stringer, Ana V. Pascual-Linaza, David Corbett, Suzan Thompson, Mark E. Arnold

**Affiliations:** 1 Animal and Plant Health Agency, Woodham Lane, United Kingdom; 2 French Agency for Food, Environmental and Occupational Health & Safety, France; 3 Department of Agriculture, Environment and Rural Affairs, Veterinary Epidemiology Unit, Belfast , Northern Ireland; 4 Veterinary Sciences Division, Bacteriology Branch, Agri-Food and Biosciences Institute, Belfast, Northern Ireland; 5 Animal and Plant Health Agency Sutton Bonington, Sutton Bonington, Loughborough, England; University of Messina: Universita degli Studi di Messina, ITALY

## Abstract

In the UK and Ireland, the European badger is the main wildlife reservoir for *Mycobacterium bovis* (*M. bovis*), the causal agent of bovine tuberculosis (bTB). The ability to diagnose *M. bovis* infection in badgers is critical to understanding the epidemiology of the infection in this species and for informing control strategies. In this study we determined the sensitivity and specificity of a lateral flow assay (Dual Path Platform (DPP) VetTB assay) to identify infected live badgers using two blood sample types: fresh whole blood (suitable for immediate testing in the field without further processing) and serum (which can be stored frozen for batch testing). Two measures were used for the interpretation of test results: qualitative visual interpretation and quantitative measurement using an optical reader for a range of cut-offs. To overcome the absence of a gold standard comparison test, we used Bayesian latent class methods, applied to results from different sub-populations. Regardless of sample type, the highest sensitivity and specificity of the DPP under qualitative interpretation were obtained using Band 1 (MPB83 antigen) results. Median estimates (95% CIs) of sensitivity and specificity were 79.9% (66.1–91.4%) and 93.3% (90.7–95.7%), respectively for whole blood and 53.0% (43.0–63.7%) and 96.3% (94.7–97.7%), respectively for serum. Band 2 (ESAT-6/CFP-10), when interpreted on its own, had median sensitivity estimates of 21.4% (12.0–32.4%) for whole blood, and 6.8% (3.3–11.9%) for serum. When using Band 1 results from the optical reader, the estimate of sensitivity for whole blood was higher than for serum across the whole range of cut-offs, though with a concomitant reduction in specificity. This study provides reliable estimates of test characteristics for the DPP when applied to whole blood and serum. The results support the use of the DPP test in a field application to identify infected live badgers using whole blood samples.

## Introduction

In the UK and Ireland, the European badger (*Meles meles*) is the main wildlife reservoir for *Mycobacterium bovis* (*M. bovis*), the main causal pathogen of bovine tuberculosis (bTB) [1,2]. Diagnosis of *M. bovis* infection in badgers is key to understanding the epidemiology of infection in this species, and to informing effective disease control, either at the individual level (e.g., to inform the targeted removal of infected animals) or at the population level (e.g., to estimate changes in infection prevalence in relation to a particular control intervention) [3]. However, accurate diagnosis of tuberculosis is challenging in all species. In badgers, mycobacterial culture or molecular detection of *M. bovis* from *post-mortem* tissues are the most established diagnostic method and, combined with genetic typing, have the advantage of near perfect specificity (>99%) [4], and high sensitivity/low limit of detection, depending on a number of diagnostic factors (number/size of tissues, culture methodology, carcass condition etc.) [4,5]. However, this approach is relevant only after the death of the animal in order to obtain tissues where *M. bovis* can be detected. Clinical samples can be derived from live, anaesthetised animals (e.g., urine, faeces, tracheal aspirate and wound exudate) [5] but culture of these provides very low diagnostic sensitivity [6,7]. Regardless of sample type, the use of mycobacterial detection to diagnose infection requires access to specialist expertise and high containment facilities and takes a significant period of time to complete (a minimum of 8 weeks as standard) and incurs substantial cost. This greatly limits its usefulness as an operational tool for disease control.

Blood-based immunological assays offer a practical and potentially more sensitive diagnostic approach. Blood can be obtained from live animals (anaesthetised or conscious) [8], and immunological assays are faster and significantly cheaper than culture. Serial blood samples are particularly valuable in field studies where longitudinal testing of cohorts can be achieved, for example in vaccine field trials [9,10].

Immunological tests for the detection of *M. bovis* infection in badgers include an interferon gamma release assay (IGRA) [11] and two serological assays: the now discontinued Brock TB Stat-Pak test (Stat-Pak, Chembio Diagnostics Systems Inc., Medford, New York 11763, USA), and its replacement, the DPP^®^ VetTB assay (referred to as DPP hereafter, also manufactured by Chembio Diagnostics Systems) which is the subject of this paper.

The DPP is a lateral-flow assay which detects immunoglobulin G (IgG) antibodies against the *Mycobacterium tuberculosis* complex antigens MPB83 and ESAT-6/CFP10 of multiple species infected with *M. bovis* [12–14]. It is suitable for use in the field with low badger sample volume (10μl of whole blood, or 30μl of serum)[12], where a rapid result is needed (result in ~ 20 minutes), and has the option of qualitative or quantitative interpretation (i.e., by eye; or using a densitometric reader). This enables timely disease control decision making and could offer potential for trap-side testing (using whole blood only).

Initial evaluations of the DPP test for the detection of *M. bovis* infection in badgers have been published [12,15]. The estimates of DPP diagnostic test performance generated by these studies were based on the performance of DPP relative to established diagnostic measures in field badgers and in experimentally infected (the infection of which was certain) or naïve captive badgers. For field evaluation, the assumption that the tests (or combination of tests) to which DPP was compared were a true gold standard could have led to bias in sensitivity and specificity estimates. Given the absence of gold standard test, the aim of the present study was to determine the performance of the DPP test to detect infection in live badgers using Bayesian latent class modelling (BLCM) [16]. Bayesian models combine prior assumptions for unknown parameters with maximum likelihood inference on the available data to provide final (posterior) estimates which are a weighted average of the prior assumptions and inference from the data. The method is suitable for evaluating diagnostic test performance and disease prevalence without requiring a gold standard [16] and has been used previously to estimate the diagnostic accuracy of established live badger tests [7,17].

As in the published studies [12,15], we estimated qualitative and quantitative test interpretation for serum and whole blood samples. We used results from different populations (captive *M. bovis* free badgers and groups trapped at different locations in the wild) and *a priori* information of other tests (IGRA and culture) performed on the same animals.

## Materials and methods

### Study populations

This study utilised data from three distinct sub-populations of badgers which were sampled between 2014 and 2016 (and grouped by year in the analysis):

The captive *M. bovis* free group, referred to as NEC (Natural Environment Centre), consisted of captive badgers sourced from low bTB risk areas of England with no history of infection in wild badgers or in local cattle. These animals were confirmed to be *M. bovis* free before inclusion, as previously described [12,18–20], and provided an *M. bovis* naïve population.

We also used data from two free-living badger populations with endemic *M. bovis* infection. These populations are referred to as Woodchester Park (WP) and Northern Ireland (NI). The data from NI has been published previously [21], whereas the data from WP was collected for the present study, although a subset of this data has previously been analysed using a different approach [12].

Animals in the WP population were sampled and tested as part of a long-term epidemiological study of *M. bovis infection* in badgers [22,23]. Four cycles of trapping were carried out in each year and some individuals were therefore captured and sampled on multiple occasions.

In Northern Ireland, data were collected during the course of a test and vaccinate or remove research trial (TVR) [15]. Badgers were caught over a 100km^2^ area from July to October over two consecutive years. Trapped badgers were individually identified by microchip and sampled at first capture each year.

### Sample collection

All sampling was carried out under general anaesthesia as approved by local authorisation issued by the appropriate authorities. In England, all badgers were trapped under a under a Natural England Science and Conservation licence. Clinical samples were collected by experienced personnel holding relevant Personal Home Office licences and acting under a Home Office Project licence. All work was subject to approval by the APHA Animal Welfare and Ethical Review Body and animals were examined and sampled under the supervision of a Named Veterinary Surgeon and a Named Animal Care and Welfare Officer. The TVR Research Project operated under the Animals (Scientific Procedures) Act 1986 (as amended) issued to DAERA by the Department of Health (DoH) in Northern Ireland. Licences were also obtained from the Northern Ireland Environment Agency (NIEA) to allow the capture, sampling, collaring and removal of badgers.

Blood was collected from the jugular vein. For serum, blood was collected using serum separator vacutainer tubes (Becton, Dickinson and Company, USA). These samples were subsequently transferred to the laboratory where they were centrifuged and the serum removed, divided into small single-use aliquots and stored at −80 °C prior to testing. Whole blood samples were collected using heparinised vacutainer tubes (Becton, Dickinson and Company, USA) and were tested after collection (without freeze-thawing).

For WP and NI badgers, clinical samples for mycobacterial culture were collected concurrently. At WP, samples of faeces, urine, tracheal and oesophageal aspirate, pus from abscesses and bite wound swabs were collected for mycobacterial culture. Clinical samples from NI badgers included tracheal aspirates and nasopharyngeal swabs from all badgers and, where appropriate, swabs from bite wounds.

### Diagnostic tests

Different diagnostic tests were applied in parallel to badger samples. Firstly the interferon gamma release assay (IGRA), which detects a cell-mediated immune response to the infection, the DPP applied to serum (DPP serum) and the DPP applied to whole blood (DPP WB), which detect antibodies to *M. bovis*, and mycobacterial culture of clinical samples, which detects the live bacteria (*M. bovis*) that cause infection. By combining the results of all these tests, we aimed to use BLCMs to identify suitable cut-off points for DPP when quantitative measurement of band intensity was measured and to infer the true overall proportion of badgers infected with *M. bovis.*

Sample sizes for each test, stratified by year and population, are provided in Table 1

**Table 1:** Number of samples by diagnostic tests across sub-populations included in the model.

Dataset	Number of samples used by test				Multiple test combinations
	IGRA	DPP whole blood	DPP serum	Clinical samplesculture	
WP (2014)	222	222	222	222	222 (all 4 tests)
WP (2015)	218	30	218	218	30 (all 4 tests)
					188 (excluding DPP whole blood)
NI (2014)	273	^	273	273	273 (excluding DPP whole blood)
NI (2015)	341	341	341	341	341 (all 4 tests)
NEC (2015)	71	71	71	0	71 (excluding culture)
NEC (2016)	88	0	88	0	88 (by IGRA & DPP serum)
Total tested	1213	664	1213	1054	

WP = Woodchester Park; NI = Northern Ireland; NEC=Natural Environment Centre. ^178 DPP whole blood tested, but only overall results (Band 1 and 2 combined) available, therefore excluded from analysis.

#### DPP^®^ VetTB assay.

**Fig 1:**
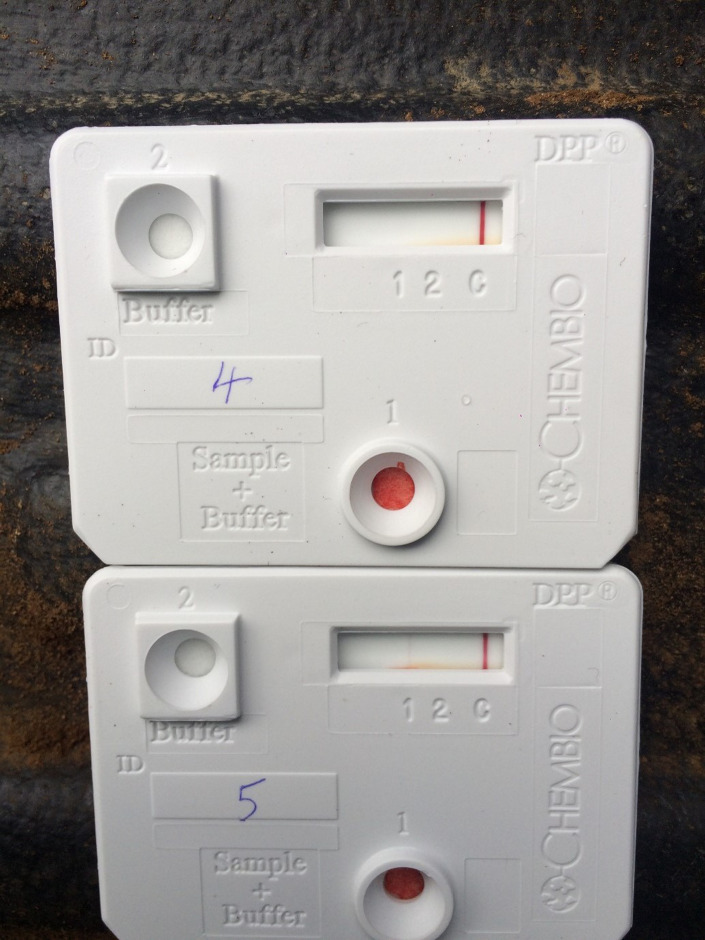
Two DPP cassette kits showing a negative result (top cassette) and a visually positive result at Band 1 (bottom cassette).

The DPP (Chembio Diagnostics Systems Inc., Medford, New York 11763, USA) has two antigen test bands (along with a positive control band) detecting antibodies to MPB83 (Band 1) and ESAT-6/CFP-10 (Band 2). Only assays with a visible control line result were used. The DPP was performed with 30 μl of serum (DPP serum) or 10 μl heparinised whole blood (DPP WB) following the protocol described in [12]. The use of 30 μl serum instead of the manufacturer’s recommended 5 μl serum followed a sample optimization study carried out by APHA (see supplementary data in [12]). During testing with DPP, the sample (serum or WB) was placed in Well 1 of the cassette along with a buffer solution (Fig 1). Further buffer was added to Well 2 five minutes later and the test was interpreted after a further 15 minutes (Fig 1). Test results were read by eye (qualitative result) and a proprietary densitometry reader supplied by the test manufacturer (DPP reader; quantitative result, expressed in relative light units (RLU)). Samples collected from NEC, NI and WP study populations were tested at these respective locations. Whole blood samples were tested directly after collection during sampling events, whilst sera were aliquoted and stored frozen prior to testing.

#### Interferon gamma release assay (IGRA).

This test was conducted with heparinised whole blood as described previously [11]. Briefly, heparinised whole blood was stimulated overnight using both *M. bovis* (PPDB) and *M. avium* (PPDA) tuberculins as antigens (Prionics Lelystad B.V., Lelystad, Netherlands). Following antigen stimulation, supernatants from the blood cultures were used to test for badger interferon gamma by ELISA [11]. Quantitative data for levels of interferon gamma produced to bovine and avian tuberculins (PPDB minus PPDA) were converted into binary test results (positive or negative) on the basis of a cut-off determined during the assay’s development [11].

#### Mycobacterial confirmatory culture of clinical samples.

Clinical samples were cultured for *M. bovis* in liquid medium BACTEC mycobacterium growth indicator tube (MGIT) 960 system (BD Diagnostics, USA) in NI, in accordance with WOAH standards [24], or for WP samples, on a modified Middlebrook7H11 agar slopes after CPC decontamination as previously described [4]. Badgers were assigned either positive or negative culture results; a positive culture result was assigned if at least one sample was positive by culture and a negative culture result when all samples cultured were negative. Cultures were further confirmed as *M. bovis* by spoligotyping [25].

### Bayesian analysis

#### Bayesian model.

The Bayesian model estimated the sensitivity and specificity of all the tests included in the model (Table 1) with data presented as positive or negative (based on visual interpretation) for each DPP test. Where both DPP serum and DPP WB had been used in parallel on the same animals, conditional dependence between these two tests was included in the model, as the results of DPP serum and DPP WB were expected to be correlated (see supplementary information for representation of correlation in the model). For all other tests, the mechanism for disease detection was different so they were considered conditionally independent. Sensitivity of culture of clinical specimens was allowed to differ between NI and WP as different tissues and culture methods were used at each location. This resulted in the following models being simultaneously fitted to the data, based on the test combinations available (Table 1): a 4-test model with the DPP tests conditionally dependent for WP data from 2014 plus samples taken from 2015 where all four tests had been applied (n = 252) and from NI from 2015 (n = 341), a 3-test model with all tests independent for WP from 2015 (n = 188) and NI (2014, n = 273), a 3-test model with two tests correlated for NEC from 2015 (n = 71), and a 2-test model with both tests independent for NEC from 2016 (n = 88). The models were based on those previously developed [16,26] (details in S1 Supporting Information). This resulted in the following parameters being estimated: sensitivity and specificity of IGRA, DPP (serum and WB), and the culture of clinical specimens (with sensitivity varying between WP and NI), plus specificity and sensitivity correlation for DPP WB and serum, and the infection prevalence of 32 populations (24 badger social groups for WP, 2 for NI (2014 and 2015), 2 for NEC (2015) and 4 for NEC (2016)).Prior information about the diagnostic tests’ sensitivity and specificity were included in the form of beta distributions (Table 2), which are commonly used to model binary variables in Bayesian analyses, since they are flexible and have a range from 0 to 1. The DPP tests were given non-informative priors in the form of beta distributions with both parameters equal to one, which provided uniform priors over the range 0–1. The sensitivity and specificity of culture of clinical samples were initially given priors based on [7]; however this resulted in a much poorer fit of the model compared to using non-informative priors, so an uninformative beta(1,1) prior was used for culture sensitivity. The model also required input of prior distribution for the prevalence of *M. bovis* infection in each sub-population. The prevalence of infection in the NEC badger population was set to a constant zero as all animals were confirmed *M. bovis* negative prior to experimental infection. The prevalence parameters for all other sub-populations were set to beta distributions with both parameters equal to 1 to provide a non-informative prior. To test the impact of the priors on the posterior estimates of the parameters, a model was also run with all parameters set to be non-informative.

**Table 2:** Prior distributions for diagnostic tests used for a Bayesian model to estimate the sensitivity and specificity of five diagnostic tests for *M. bovis* infection in live badgers.

Test	Parameter	Source of prior	Beta prior distribution	Median (2.5^th^ – 97.5^th^ percentiles)
Interferon Gamma	Se	[7]	(54.9,14.6)	0.793 (0.688–0.877)
	Sp	[7]	(222.5,12.7)	0.947 (0.914–0.971)
DPP whole blood	Se	Non-informative	(1,1)	
	Sp	Non-informative	(1,1)	
DPP serum	Se	Non-informative	(1,1)	
	Sp	Non-informative	(1,1)	
Culture (WP)	Se	Non-informative	(1,1)	
	Sp	[7]	(1050.8,3.1)	0.997 (0.993–0.999)
Culture (NI)	Se	Non-informative	(1,1)	
	Sp	[7]	(1050.8,3.1)	0.997 (0.993–0.999)

#### Software.

Estimation of the unknown parameters was carried out using WinBUGS 14 [27], controlled by Stata 14 [28]. This approach works by having initial starting values, which converge towards the final (posterior) estimates and so the first 5,000 iterations were discarded as a burn-in period before convergence had been reached. After the burn-in period, 10,000 iterations were used to generate the final (posterior) estimates. Three chains with different initial values were run for each model and convergence was checked by visual inspection of plots of variable values and by use of the Gelman-Rubin convergence statistic, as implemented in WinBUGS [29].

In order to assess the relative performance of the DPP for different antigens for both WB and serum, we evaluated four scenarios: (I) visual interpretation of Band 1 only (II) visual interpretation of Band 2 only, (III) visual interpretation of both Band 1 and Band 2 in parallel, and (IV) quantitative interpretation of Band 1. The quantitative response for Band 2 was not evaluated given the poor performance of scenario II (see results).

#### Model fit.

The fit of each model to the data was assessed by using Bayesian *p*-values. In short, the method looks at how closely data simulated using the posterior estimates matches the observed data, and calculates a p-value based on Pearson chi-squared statistics. In this case, either a very low or very high p-value represents a poor fit of the model to the data.

The WinBUGS code for the evaluation of model fit was adapted from existing WinBUGS code [30].

#### Impact of different DPP cut-off values.

For the quantitative DPP values (measured in relative light units (RLUs)), estimates of sensitivity and specificity at different RLU cut-offs were produced by re-evaluating which DPP samples were positive at the given cut-off, and refitting the BLCM. The analysis was conducted for DPP WB and DPP serum independently: new positive/negative data were analysed for DPP WB while using DPP serum visual interpretation data, and then the process was reversed for DPP serum assessment. This process was repeated for cut-offs across the range of possible values.

## Results

### DPP sensitivity and specificity based on visual interpretation

**Fig 2:**
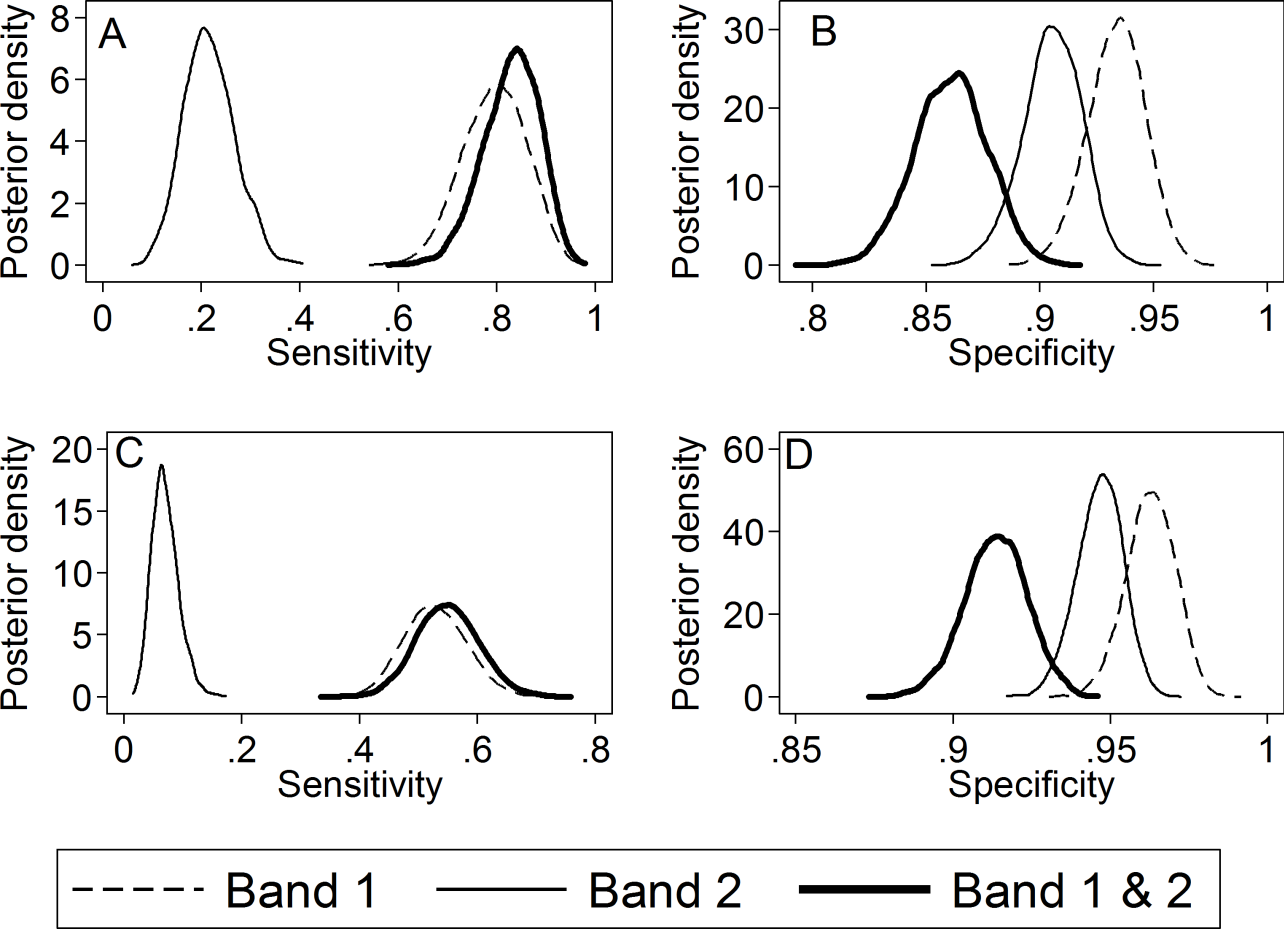
Posterior density of DPP diagnostic test performance to detect *M. bovis* infection in live badgers under use of Band 1 only, Band 2 only, and both Bands in parallel for A) sensitivity when applied to whole blood B) specificity when applied to whole blood C) sensitivity when applied to serum D) specificity when applied to serum.

The highest sensitivity and specificity of the DPP interpreted visually were obtained with scenario I (using Band 1 (MPB83)) either with WB or serum. Median estimates (95% CI) of the sensitivity and specificity were 79.9% (66.1–91.4%) and 93.3% (90.7–95.7%), respectively for DPP WB and 53.0% (43.0–63.7%) and 96.3% (94.7–97.7%), respectively for DPP serum (Table 3, Fig 2A and 2C; and Tables S1 and S2, Figs S1 and S2 in S2 Appendix). DPP using Band 2 only (ESAT6/CFP-10; scenario II) had sensitivity estimate of 21.4% (12.0–32.4%) and specificity of 90.4% (87.8–92.9%) for WB, and sensitivity of 6.8% (3.3–11.9%) and specificity of 94.7% (93.1%-96.0%) for serum) (Fig 2A and 2C, Tables S1 and S2 in S2 Appendix). While scenario (III) (i.e., positive if either band visible) resulted in a slightly higher sensitivity than scenario (I) (median of 83.5% and 55.2% for WB and serum respectively compared to 79.9% and 53.0% for scenario I)it resulted in much lower specificity estimates (85.9% (82.7–89.2) for WB and 91.4% (89.3–93.3%) for serum (Fig 2B and D, Tables S1 and S2 in S2 Appendix). IGRA had a median sensitivity of 66.4%, which was higher than DPP serum (53.0%) but a poorer sensitivity than DPP WB (79.9%). The median estimate of IGRA specificity was higher (96.9%) than for either DPP WB (93.3%) or DPP serum (96.3%). Mycobacterial culture of clinical samples had a low sensitivity relative to the other tests (medians (95% CI) of 17.9% (11.0 – 26.6%) and 34.8% (20.3 – 53.9%) for WP and NI, respectively), but a very high specificity (medians > 99%) (Table 3).

**Table 3:** Posterior distributions from a Bayesian model to estimate diagnostic test sensitivity and specificity for *M. bovis* infection in live badgers.

	Sensitivity		Specificity	
	Median	95% CI**	Median	95% CI**
DPP WB*	0.799	0.661–0.914	0.933	0.907–0.957
DPP serum*	0.530	0.430–0.637	0.963	0.947–0.977
IGRA	0.664	0.576–0.750	0.969	0.955–0.981
Culture WP	0.179	0.110–0.266	0.996	0.992–0.999
Culture NI	0.348	0.203–0.539	0.998	0.994–0.999

*Scenario I (Band 1 for MPB83 present, independently from Band 2) with a visual interpretation.

**CI = Credible Interval.

Important conditional dependence between both the sensitivity and specificity of DPP WB and DPP serum was observed (parameters ρ_1_ and ρ_2_ representing this (see Supplementary Information) was given by ρ_1_ = 0.06 (95% CrI: 0.003 – 0.13)), and ρ_2_ = 0.02 (95% CrI: 0.01 – 0.034)).

The model also provided estimates of infection prevalence for NI (5.7% (3.5–8.9%) in 2014 and 2015, and WP by social group (range from 2.6–71.9%, mean of 28.6%, Table S4 in S2 Appendix). Importantly, if non-informative priors were used instead of beta prior estimates listed in Table 2, the estimates of sensitivity for both DPP WB and DPP serum reduced by 7–8% (Table S3 in S2 Appendix compared to Table 3) to approximately 72% and 46%, respectively. The sensitivity of IGRA was reduced even further, by approximately 14% (from 66% to 52%). The specificity of the DPP tests and IGRA was also reduced by around 1–2%. Culture also showed a reduction in sensitivity estimates using these priors, but specificity was reduced by less than 1%.

Based on scenario I, the DPP WB produced a consistently higher proportion of positive results (29% and 9% for WP and NI, respectively) compared with IGRA (18% and 8% for NI and WP, respectively), DPP serum (15% and 8% for WP and NI, respectively) and culture of clinical samples (5% and 3%) for NI and WP, respectively (Table 4). Looking at the totals for WP and NI by year, in the years where there were many samples of DPP WB, serum and IGRA (WP 2014 and NI 2015), there appeared to be a consistent pattern of DPP WB having the most positives, and similar numbers of IGRA and DPP serum positives. In WP in 2015, there was a different pattern, with fewer DPP serum than IGRA (Table 4). In that year, there appeared to be a greater number of DPP serum positives that were negative for IGRA, and a greater number of IGRA positives that were negative for DPP serum (Tables S5 and S6 in S2 Appendix). Among the bTB free badgers (NEC), 4% were falsely identified as positive using DPP WB and 3% for DPP serum (Table 4).

**Table 4:** The number of positive tests in each data set included in a Bayesian model to evaluate the sensitivity and specificity of diagnostic tests for *M. bovis* infection in live badgers.

Dataset	Number of animals positive/ total tested (% positive)			
	IGRA	DPP WB: Band 1 only	DPP serum: Band 1 only	Clinical samples culture
WP 2014	41/222 (19%)	70/222 (32%)	48/222 (22%)	18/222 (8%)
WP 2015	36/218 (17%)	3/30 (10%)	18/218 (8%)	3/218 (1%)
**WP Total**	**77/440 (18%)**	**73/252 (29%)**	**66/440 (15%)**	**21/440 (5%)**
NI 2014	26/273 (10%)		28/273 (10%)	11/273 (4%)
NI 2015	22/341 (6%)	32/341 (9%)	24/341 (7%)	7/341 (2%)
**Ni Total**	**48/614 (8%)**	**32/341 (9%)**	**52/614 (8%)**	**18/614 (3%)**
**NEC**	**0/159 (0)**	**3/71 (4%)**	**5/159 (3%)**	**N/A** ^a^
Total positive	125	108	123	39
Total tested	1213	664	1213	1054

WP=Woodchester Park; NI=Northern Ireland; NEC=Natural Environment Centre; N/A = Not applicable.

^a^Note: all NEC clinical samples tested by culture were negative and not included in the analysis.

### Model fit

The model appeared to be a good fit to the data, with Bayesian *p*-values ranging from 0.23–0.77 (Table S7 in S2 Appendix).

### DPP reader cut-off points

**Fig 3:**
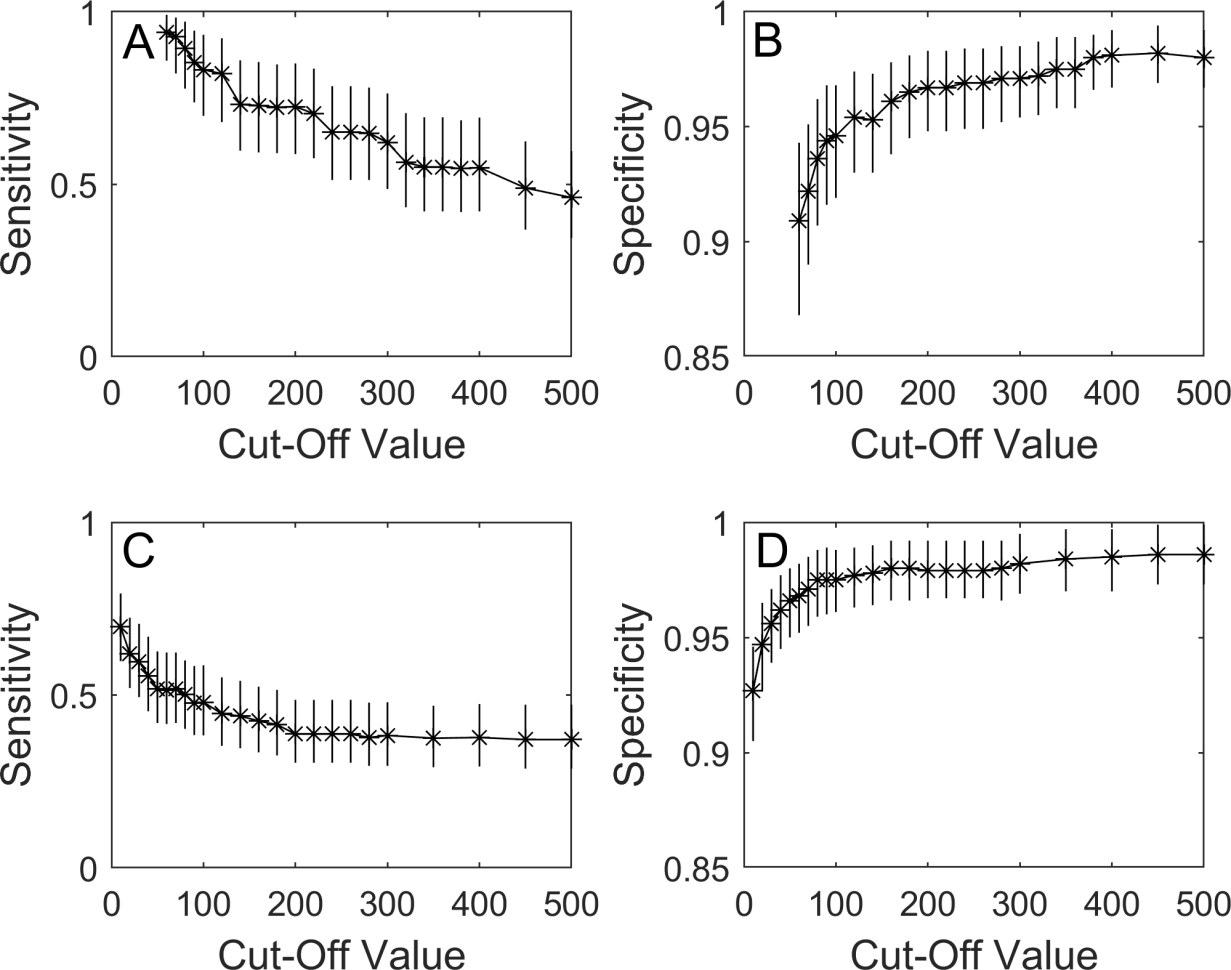
Posterior median from a Bayesian model (with points marked by asterisks) and 95% CrI sensitivity and specificity (vertical bars) of DPP test performance to detect *M. bovis* infection in live badgers. Estimates are from a range of different cut-off thresholds to classify a sample as positive, with estimates are given for A) DPP sensitivity when applied to whole blood B) DPP specificity when applied to whole blood C) DPP sensitivity when applied to serum D) DPP specificity when applied to serum.

The median estimates of sensitivity for DPP WB when read electronically were higher than for DPP serum across the whole range of different cut-offs for designating a sample as positive (Fig 3A, 3C; Tables S8, S9 in S2 Appendix). The high sensitivity shown at low RLU cut-offs corresponded with low specificity levels (Fig 3B, 3D; Tables S8, S9 in S2 Appendix). The DPP reader interpretation also performed slightly better than the visual interpretation. For example, the cut-off point of 80 RLU for DPP WB gave both a higher sensitivity (89.4% DPP reader compared to 79.9% visual interpretation) and specificity (93.6% DPP reader compared to 93.3% visual interpretation). Results for DPP WB for RLU < 60 had poor fit to the data (Bayesian p-value < 0.02), so values below that range were not considered to be robust and were not reported.

At a cut-off of 40 RLU for DPP serum, which gave a similar median specificity as for DPP serum by visual interpretation (96.2% and 96.3%, respectively), a median sensitivity of 55.6% was obtained by the electronic reader, compared to 53.0% from the DPP serum visual interpretation.

## Discussion

In this study we determined the sensitivity and specificity of the DPP test to identify *M. bovis* infected live badgers for two blood sample types: fresh whole blood (suitable for immediate testing in the field without further processing) and serum (which can be stored frozen for batch testing). We used two outcome measures: binary (positive/negative) based on the visual (qualitative) observation of up to two results (Band 1 and Band 2), and continuous (quantitative) measurement of band intensity using an electronic reader which also allowed the calculation of cut-off points.

To overcome the lack of gold standard for *M. bovis* infection at post-mortem [4] which introduces bias in the calculation of test sensitivity and specificity, we used BLCMs, results from different populations (captive with known *M. bovis* (bTB) free status and badger groups trapped at different locations in wild TB endemic areas) and *a priori* information of the (imperfect) tests performed on the same animals.

The findings of the present study are consistent with that of an earlier study [12], which evaluated DPP in terms of relative sensitivity to other tests (i.e., using a pseudo-gold standard approach). Using the visual interpretation of the test (which is more practical for trap-side use), and interpreting the DPP test using band 1 only, this earlier study showed that, based on serum from culture-confirmed naturally infected and *M. bovis* free captive badgers, the sensitivity was 55.3% (95% CI: 39.5 to 71.1) at a specificity of 92.5% (95% CI: 85.3 to 99.6). While the sensitivity estimate is similar to that for the present study, the specificity was almost 4% lower, which may result from the estimation of relative sensitivity rather than applying a latent class model. In other words, the assumption that post mortem was a gold standard may have led to any positive DPP serum samples that were negative for post mortem being wrongly assumed to being a false positive, which may not be true of all such samples, leading to a lower estimate of specificity. The use of a more extensive data set also reduced the width of the uncertainty interval around the sensitivity estimate. In both analysis, Band 1 data were more informative, in contrast to Band 2 which provided less sensitive and less specific diagnostic information [12]. This not a surprising finding given that the antigen present at Band 1 (MPB83) is known to the dominant antigen to which badgers produce an immunological response following *M. bovis* infection while responses to ESAT-6/CFP-10 are more variable [13].

There was also a difference in the estimate of sensitivity of DPP WB between the present study and the earlier one, where the sensitivity of DPP WB was 65% compared to 79% in the present study. One reason for the difference was the use of informative priors, particularly for IGRA sensitivity, which resulted in higher estimates for the sensitivity of the tests compared to non-informative priors (Table S3), although the use of latent class methods in place of pseudo gold-standard approaches and the differences in the data sets would also have contributed to the difference in estimates. However, there is agreement between the two studies in the finding of a higher sensitivity using DPP WB than using serum samples from the same badger. One possible explanation for the lower sensitivity observed using serum might be if DPP serum produced a prozone-like effect through the saturation of the membrane with antibodies [31] in serum samples of 30 μl instead of 10 μl for WB. A prozone effect occurs where there is an excess of antibodies that then prevent binding with the antigen inhibiting the formation of immune complexes and thus causing false-negative results in a serological test. However, we do not believe this accounts for the observed difference, as the sample volume of serum was optimised for use in badgers at 30 μl rather than 5 μl used in other species (see supplementary data provided in [12]). The high level of positivity obtained in the Bayesian analysis for DPP WB may be the reflection of non-specific reactivity to the DPP antigens (e.g., due to exposure to environmental mycobacteria) as 9.9% of animals from the same site at Woodchester Park were positive by DPP WB but negative by all other tests. However, it is difficult to be certain of the absence of an underlying *M. bovis* infection in absence of reliable gold standard diagnostic in these animals. Moreover, a similar response would have been expected with the DPP serum test.

Sensitivity of serological tests tends to be higher in animals with more advanced disease [32], which is valuable in targeting those most at risk of transmitting the infection. Excretion has also been shown as early 4 weeks after experimental infection in badgers [19], when serological responses measurable by DPP also tend to increase [12]. This study used samples from two naturally infected populations in areas of endemic bTB infection prior to any disease control intervention. The infection prevalence in the WP population was estimated to be much higher than that of the NI one, which could lead to differences in test performance between the populations, and potentially a higher sensitivity of DPP in WP compared to NI. There was little evidence of this though, as the pattern of positive DPP/IGRA tests was very similar between WP and NI, apart from WP 2015 where few DPP WB samples were taken. Consequently, our sample likely represents a wide range of different stages of infection and pathology and the results thus provide a useful population-level estimate of test performance.

Estimates of DPP test sensitivity for visual and quantitative interpretations were broadly consistent at equivalent specificities. The use of the electronic reader is being developed for laboratory processing of larger batches of samples, to quantify the results and reduce the potential for inter-observer and inter-location (either in the laboratories or in the field) variations in test results [15]. Using the electronic reader, lower cut-off points could be applied to increase sensitivity (at the expense of the specificity) which may be of value towards the later stages of bTB eradication. However, the high cost and limited availability of electronic readers mean visual interpretation of the test results is more practical in the field situation.

While not a primary objective of the study, the BLCM provided estimates of the diagnostic performance of the other tests included in the datasets. The estimated characteristics of these other diagnostic tests were broadly consistent with previous models. The present study estimated a median sensitivity of the IGRA test of 66.4%, which falls between previous estimates of 79.9% (95% CI 68.8–89.5%) [7] and 52% (95% CI 46–63%) [17], but which is significantly lower than the original (relative) sensitivity estimate of 80.9% [11].

The low sensitivity and high specificity of culture of clinical samples was expected [33], although the estimates of sensitivity from the present study (17.9% for WP, 34.8% for NI) were higher than the 4.1–8% previously reported in free-living badgers [7,10,17].

The DPP assessment relative to established diagnostic tests [15] in the NI badger population provided lower estimates of sensitivity of DPP WB and serum (Band 1 only) (50% for WB and 42% for serum) compared to the current study using the first-capture badger data (n = 456) from the first two years of the TVR project [34]. These sensitivity estimate differences may have arisen in part due to the assumption that culture/IGRA applied in parallel were a gold standard. This paper also found that the use of Band 1 only provided the best sensitivity, and the specificity estimates (95% WB, 96% serum) within the 95% CIs obtained for the current study’s DPP WB and DPP serum estimates. The variability in inter-rater performance shown by [15] may also explain the slightly better performance of the DPP reader quantitative assessment over qualitative visual assessment.

A similar Bayesian model was also applied to the five years (2014–2018) of TVR data obtained from NI [21]. Results were generally comparable to those found in the present analysis, although DPP WB sensitivity was higher in the present study (posterior median of 79.9% compared to 69% [21]) and DPP WB specificity was estimated to be lower in the present study (posterior median of 93.3% compared to 97% [21]). However, the removal of DPP positive badgers and badger vaccination only occurred in the TVR, which may have contributed to any differences between the two studies, as well as the use of informative priors for the sensitivity and specificity of IGRA in the present study.

In conclusion, this study provides reliable estimates of test characteristics for the DPP using robust statistical methods appropriate to evaluating test performance in the absence of a gold standard comparison test. The high sensitivity observed with whole blood samples supports field application and demonstrates the potential for use of the test in surveillance and operational scenarios. The use of an electronic reader has the potential to increase sensitivity and objectivity of the test evaluation.

## Supporting information

Appendix S1 Model equations. (DOCX)

Appendix S2 Additional tables and figures. (DOCX)

Appendix S3 NEC data. (XLSX)

Appendix S4 WP data. (XLS)

Appendix S5 NI data. (XLSX)
