# Supplementary material for: Evaluating sensitivity and specificity of the DPP Vet TB assay in badgers using Bayesian latent class models
Source: PLoS One. 2025 Mar 4;20(3):e0313825. doi: 10.1371/journal.pone.0313825 (PMC11878917; doi:10.1371/journal.pone.0313825)
Supplement: Appendix S1 — (DOCX) [file pone.0313825.s001.docx]

**Supporting Information for Evaluating sensitivity and specificity of the DPP Vet TB assay in badgers using Bayesian latent class models**

Rachel C. Jinks, Sandrine Lesellier, Freya Smith, Fraser D. Menzies, Roland T. Ashford, Laura Waring, Dipesh Dave, Paul Anderson, Lesley A. Stringer, Ana V. Pascual-Linaza, David Corbett , Suzan Thompson , Mark E. Arnold.

**Appendix S1 Model Equations**

Denote the sensitivity of IGRA, DPP WB, DPP serum and culture of clinical samples by *Se_1_, Se_2_, Se_3_, and Se_4_* respectively and similarly the specificity of each test by *Sp_1_, Sp_2_, Sp_3_, and Sp_4.._* The test data arise from a multinomial distribution with 16 possible outcomes, and we denote the outcomes of each of the test by T_1,_ T_2,_ T_3_ and T_4_ respectively, with a suffix of +/- to denote whether the outcome was positive or negative. The DPP test results with WB/serum are possibly correlated, and so conditional dependence is included by ρ_1_ and ρ_2_ for the sensitivity/specificity respectively. Denoting further the infection prevalence in sub-population *i* by *π_i_* the cell probabilities for each outcome are given by(Enoe et al., 2000; Branscum et al., 2005) .

$$P\left( T_{1}^{+}T_{2}^{+}T_{3}^{+}T_{4}^{+} \right)=\pi_{i}Se_{1}\left( Se_{2}Se_{3}+\rho_{1} \right)Se_{4}+(1-\pi_{i})(1-Sp_{1})(\left( 1-Sp_{2} \right)\left( 1-Sp_{3} \right)+\rho_{2})(1-Sp_{4})$$

$$P\left( T_{1}^{+}T_{2}^{+}T_{3}^{+}T_{4}^{-} \right)=\pi_{i}Se_{1}\left( Se_{2}Se_{3}+\rho_{1} \right)\left( 1-Se_{4} \right)+\left( 1-\pi_{i} \right)\left( 1-Sp_{1} \right)\left( \left( 1-Sp_{2} \right)\left( 1-Sp_{3} \right)+\rho_{2} \right)Sp_{4}$$

$$P\left( T_{1}^{+}T_{2}^{+}T_{3}^{-}T_{4}^{+} \right)=\pi_{i}Se_{1}\left( Se_{2}(1-Se_{3}){-\rho}_{1} \right)Se_{4}+(1-\pi_{i})(1-Sp_{1})(\left( 1-Sp_{2} \right)Sp_{3}-\rho_{2})(1-Sp_{4})$$

$$P\left( T_{1}^{+}T_{2}^{+}T_{3}^{-}T_{4}^{-} \right)=\pi_{i}Se_{1}\left( Se_{2}\left( 1-Se_{3} \right)-\rho_{1} \right)\left( 1-Se_{4} \right)+\left( 1-\pi_{i} \right)\left( 1-Sp_{1} \right)\left( \left( 1-Sp_{2} \right)Sp_{3}-\rho_{2} \right)Sp_{4}$$

$$P\left( T_{1}^{+}T_{2}^{-}T_{3}^{+}T_{4}^{+} \right)=\pi_{i}Se_{1}\left( (1-Se_{2})Se_{3}-\rho_{1} \right)Se_{4}+(1-\pi_{i})(1-Sp_{1})(Sp_{2}\left( 1-Sp_{3} \right)-\rho_{2})(1-Sp_{4})$$

$$P\left( T_{1}^{+}T_{2}^{-}T_{3}^{+}T_{4}^{-} \right)=\pi_{i}Se_{1}\left( \left( 1-Se_{2} \right)Se_{3}-\rho_{1} \right)(1-Se_{4})+(1-\pi_{i})(1-Sp_{1})(Sp_{2}\left( 1-Sp_{3} \right)-\rho_{2})Sp_{4}$$

$$P\left( T_{1}^{+}T_{2}^{-}T_{3}^{-}T_{4}^{+} \right)=\pi_{i}Se_{1}\left( (1-Se_{2})(1-Se_{3}){+\rho}_{1} \right)Se_{4}+\left( 1-\pi_{i} \right)\left( 1-Sp_{1} \right)(Sp_{2}Sp_{3}+\rho_{2})(1-Sp_{4})$$

$$P\left( T_{1}^{+}T_{2}^{-}T_{3}^{-}T_{4}^{-} \right)=\pi_{i}Se_{1}\left( \left( 1-Se_{2} \right)\left( 1-Se_{3} \right)+\rho_{1} \right)\left( 1-Se_{4} \right)+\left( 1-\pi_{i} \right)\left( 1-Sp_{1} \right)\left( Sp_{2}Sp_{3}+\rho_{2} \right)Sp_{4}$$

$$P\left( T_{1}^{-}T_{2}^{+}T_{3}^{+}T_{4}^{+} \right)=\pi_{i}(1-Se_{1})\left( Se_{2}Se_{3}+\rho_{1} \right)Se_{4}+\left( 1-\pi_{i} \right)Sp_{1}\left( \left( 1-Sp_{2} \right)\left( 1-Sp_{3} \right)+\rho_{2} \right)(1-Sp_{4})$$

$$P\left( T_{1}^{-}T_{2}^{+}T_{3}^{+}T_{4}^{-} \right)=\pi_{i}(1-Se_{1})\left( Se_{2}Se_{3}+\rho_{1} \right)\left( 1-Se_{4} \right)+\left( 1-\pi_{i} \right)Sp_{1}\left( \left( 1-Sp_{2} \right)\left( 1-Sp_{3} \right)+\rho_{2} \right)Sp_{4}$$

$$P\left( T_{1}^{-}T_{2}^{+}T_{3}^{-}T_{4}^{+} \right)=\pi_{i}(1-Se_{1})\left( Se_{2}\left( 1-Se_{3} \right){-\rho}_{1} \right)Se_{4}+\left( 1-\pi_{i} \right)Sp_{1}(\left( 1-Sp_{2} \right)Sp_{3}-\rho_{2})(1-Sp_{4})$$

$$P\left( T_{1}^{-}T_{2}^{+}T_{3}^{-}T_{4}^{-} \right)=\pi_{i}(1-Se_{1})\left( Se_{2}\left( 1-Se_{3} \right)-\rho_{1} \right)\left( 1-Se_{4} \right)+\left( 1-\pi_{i} \right)Sp_{1}\left( \left( 1-Sp_{2} \right)Sp_{3}-\rho_{2} \right)Sp_{4}$$

$$P\left( T_{1}^{-}T_{2}^{-}T_{3}^{+}T_{4}^{+} \right)=\pi_{i}(1-Se_{1})\left( \left( 1-Se_{2} \right)Se_{3}-\rho_{1} \right)Se_{4}+\left( 1-\pi_{i} \right)Sp_{1}(Sp_{2}\left( 1-Sp_{3} \right)-\rho_{2})(1-Sp_{4})$$

$$P\left( T_{1}^{-}T_{2}^{-}T_{3}^{+}T_{4}^{-} \right)=\pi_{i}(1-Se_{1})\left( \left( 1-Se_{2} \right)Se_{3}{-\rho}_{1} \right)(1-Se_{4})+\left( 1-\pi_{i} \right)Sp_{1}(Sp_{2}\left( 1-Sp_{3} \right)-\rho_{2})Sp_{4}$$

$$P\left( T_{1}^{-}T_{2}^{-}T_{3}^{-}T_{4}^{+} \right)=\pi_{i}\left( 1-Se_{1} \right)\left( (1-Se_{2})(1-Se_{3})+\rho_{1} \right)Se_{4}+\left( 1-\pi_{i} \right)Sp_{1}(Sp_{2}Sp_{3}+\rho_{2})(1-Sp_{4})$$

$$P\left( T_{1}^{-}T_{2}^{-}T_{3}^{-}T_{4}^{-} \right)=\pi_{i}(1-Se_{1})\left( (1-Se_{2})(1-Se_{3})+\rho_{1} \right)(1-Se_{4})+\left( 1-\pi_{i} \right)Sp_{1}(Sp_{2}Sp_{3}+\rho_{2})Sp_{4}$$

The parameters determining the correlation between the DPP tests, ρ_1_,ρ_2,_ are sampled from uniform distributions such that the joint probability of the result of both tests is constrained to be between 0 and 1. So, in line with(Dendukuri and Joseph, 2001).

ρ_1~_uniform((*Se_2_*-1)(1- *Se_3_*), min(*Se_2_*,*Se_3_*) – *Se_2_***Se_3_*))

ρ_2~_uniform((*Sp_2_*-1)(1- *Sp_3_*), min(*Sp_2_*,*Sp_3_*) – *Sp_2_***Sp_3_*))

The two- and three-test models fitted to the data follow a similar pattern, with their being only 4 and 8 possible test combinations respectively. Models without conditional dependence will have ρ_1,_ ρ_2_=0.

Branscum AJ, Gardner IA, Johnson WO. Estimation of diagnostic-test sensitivity and specificity through Bayesian modeling. Prev Vet Med. 2005;68(2-4):145-63.

Enoe C, Georgiadis MP, Johnson WO. Estimation of sensitivity and specificity of diagnostic tests and disease prevalence when the true disease state is unknown. Prev Vet Med. 2000;45(1-2):61-81.

Dendukuri N, Joseph L. Bayesian approaches to modeling the conditional dependence between multiple diagnostic tests. Biometrics 2001*;* 57**:** 158-167.
