# Supplementary material for: Evaluating sensitivity and specificity of the DPP Vet TB assay in badgers using Bayesian latent class models
Source: PLoS One. 2025 Mar 4;20(3):e0313825. doi: 10.1371/journal.pone.0313825 (PMC11878917; doi:10.1371/journal.pone.0313825)
Supplement: Appendix S2 — (DOCX) [file pone.0313825.s002.docx]

**Supporting Information for Evaluating sensitivity and specificity of the DPP Vet TB assay in badgers using Bayesian latent class models**

Rachel C. Jinks, Sandrine Lesellier, Freya Smith, Fraser D. Menzies, Roland T. Ashford, Laura Waring, Dipesh Dave, Paul Anderson, Lesley A. Stringer, Ana V. Pascual-Linaza, David Corbett , Suzan Thompson , Mark E. Arnold.

**Appendix S2. Additional Tables and Figures.**

**DPP band selection**

Rerunning the final models using only Band 2 test result, and Band 1 and 2 in parallel, showed that using the Band 1 test result alone was the best option (Table S1). Although sensitivity for Band 1 & 2 together was higher by 3-4% for both blood and serum, specificity was markedly lower for Band 1 & 2 together than for Band 1 alone. Band 2 alone showed much lower sensitivity for both blood and serum than the other Band combinations.

Table S1.

Median (95% CI) posterior density for sensitivity and specificity of DPP whole blood test for detection of *M. bovis* infection in live badgers using only Band 1 result, only Band 2 result, or Band 1 and 2 in parallel.

|  | **Sensitivity** | **Specificity** |
| --- | --- | --- |
| **Band 1 only** | 0.799 (0.661-0.914) | 0.933 (0.907-0.957) |
| **Band 2 only** | 0.214 (0.120-0.324) | 0.906 (0.878-0.929) |
| **Band 1 & 2** | 0.835 (0.720-0.935) | 0.859 (0.827-0.892) |

Table S2.

Median (95% CI) posterior density for sensitivity and specificity of DPP serum test for detection of *M. bovis* infection in live badgers using only Band 1 result, only Band 2 result, or Band 1 and 2 in parallel.

|  | **Sensitivity** | **Specificity** |
| --- | --- | --- |
| **Band 1 only** | 0.530 (0.430-0.637) | 0.963 (0.947-0.977) |
| **Band 2 only** | 0.068 (0.033-0.119) | 0.947 (0.931-0.960) |
| **Band 1 & 2** | 0.552 (0.447-0.656) | 0.914 (0.893-0.933) |

**Model results with non-informative priors for all parameters**

Table S3.

Posterior distributions from a Bayesian model to estimate diagnostic test sensitivity and specificity for *M. bovis* infection in live badgers where non-informative priors used for all parameters.

|  | **Sensitivity** | | **Specificity** | |
| --- | --- | --- | --- | --- |
|  | **Median** | **95% CI** | **Median** | **95% CI** |
| DPP WB | 0.716 | 0.575 - 0.860 | 0.949 | 0.922 - 0.973 |
| DPP serum* | 0.457 | 0.361 - 0.562 | 0.971 | 0.955 - 0.985 |
| Gamma | 0.524 | 0.423 - 0.632 | 0.984 | 0.966 - 0.998 |
| Culture WP | 0.146 | 0.087 - 0.217 | 0.989 | 0.973 - 0.998 |
| Culture NI | 0.250 | 0.137 - 0.406 | 0.996 | 0.988 - 1.000 |

**Infection prevalence estimates**

Table S4 : Estimates of M bovis infection prevalence by badger social group in Woodchester Park, inferred from a Bayesian latent class model.

| Social group | Bayesian estimates of infection prevalence | |
| --- | --- | --- |
|  | Median | 95% Credible Interval |
| 1 | 0.156 | (0.038, 0.365) |
| 2 | 0.719 | (0.378, 0.963) |
| 3 | 0.364 | (0.125, 0.649) |
| 4 | 0.286 | (0.101, 0.53) |
| 5 | 0.047 | (0.002, 0.222) |
| 6 | 0.278 | (0.05, 0.631) |
| 7 | 0.519 | (0.262, 0.768) |
| 8 | 0.026 | (0.001, 0.127) |
| 9 | 0.573 | (0.27, 0.862) |
| 10 | 0.424 | (0.172, 0.744) |
| 11 | 0.192 | (0.048, 0.398) |
| 12 | 0.104 | (0.004, 0.407) |
| 13 | 0.066 | (0.003, 0.3) |
| 14 | 0.272 | (0.121, 0.46) |
| 15 | 0.191 | (0.029, 0.465) |
| 16 | 0.161 | (0.01, 0.464) |
| 17 | 0.683 | (0.348, 0.938) |
| 18 | 0.648 | (0.398, 0.868) |
| 19 | 0.442 | (0.201, 0.704) |
| 20 | 0.059 | (0.002, 0.28) |
| 21 | 0.06 | (0.002, 0.289) |
| 22 | 0.056 | (0.002, 0.23) |
| 23 | 0.299 | (0.142, 0.497) |
| 24 | 0.242 | (0.012, 0.71) |

**Tables comparing IGRA with DPP**

Table S5. Test results for DPP WB and serum versus those for IGRA for WP and NI sampled and tested in 2014.

| Population | DPP Test (Band 1 only) | Result | IGRA | |
| --- | --- | --- | --- | --- |
|  |  |  | Positive | Negative |
| WP | WB | + | 30 | 43 |
|  |  | - | 11 | 168 |
|  | Serum | + | 36 | 30 |
|  |  | - | 41 | 332 |
| NI | WB | + | 12 | 20 |
|  |  | - | 10 | 299 |
|  | Serum | + | 17 | 35 |
|  |  | - | 31 | 531 |

Table S6. Test results for DPP WB and serum (interpreted using band 1 only) versus those for IGRA for WP and NI sampled and tested in 2015.

| DPP test (band 1 only) | Population | Result | IGRA | |
| --- | --- | --- | --- | --- |
|  |  |  | Positive | Negative |
| WB | WP | + | 30 (11.9%) | 43 (17.1%) |
|  |  | - | 11 (4.4%) | 168 (66.7%) |
|  | NI | + | 12 (3.5%) | 20 (5.9%) |
|  |  | - | 10 (2.9%) | 299 (87.7%) |
| Serum | WP | + | 36 (8.2%) | 30 (6.8%) |
|  |  | - | 41 (9.3%) | 332 (75.6%) |
|  | NI | + | 17 (2.8%) | 35 (5.7%) |
|  |  | - | 31 (5%) | 531 (86.5%) |

**Model Fit**

Table S7. Bayesian p values, indicating the fit of a Bayesian model estimating diagnostic test performance in the absence of a gold standard to several data sets, each with a combination of diagnostic tests applied in parallel to live badgers.

| **Data set** | **Bayesian p value** |  |
| --- | --- | --- |
| WP (2014) | 0.36 | |
| WP (2015) | 0.47 | |
| NEC (2015) | 0.62 | |
| NEC (2016) | 0.23 | |
| NI (2014) | 0.77 | |
| NI (2015) | 0.31 | |

**Cut-off analysis**

Table S8. DPP WB estimates of sensitivity and specificity with test median and 95% credible interval from posterior distributions a Bayesian model. Estimates are over a range of possible cut-off values at which to determine if a test is positive.

| **Cut-off (RLU)** | **Sensitivity** | | | **Specificity** | | |
| --- | --- | --- | --- | --- | --- | --- |
|  | **Median** | **95% CI** | | **Median** | **95% CI** | |
| 60 | 0.94 | 0.858 - | 0.991 | 0.909 | 0.868 - | 0.943 |
| 70 | 0.928 | 0.821 - | 0.983 | 0.922 | 0.89 - | 0.951 |
| 80 | 0.894 | 0.777 - | 0.971 | 0.936 | 0.907 - | 0.962 |
| 90 | 0.852 | 0.737 - | 0.945 | 0.944 | 0.916 - | 0.968 |
| 100 | 0.832 | 0.698 - | 0.933 | 0.946 | 0.919 - | 0.968 |
| 120 | 0.82 | 0.68 - | 0.923 | 0.954 | 0.93 - | 0.974 |
| 140 | 0.731 | 0.597 - | 0.859 | 0.953 | 0.93 - | 0.973 |
| 160 | 0.728 | 0.592 - | 0.854 | 0.961 | 0.938 - | 0.978 |
| 180 | 0.723 | 0.59 - | 0.847 | 0.965 | 0.945 - | 0.981 |
| 200 | 0.725 | 0.587 - | 0.85 | 0.967 | 0.948 - | 0.983 |
| 220 | 0.704 | 0.575 - | 0.835 | 0.967 | 0.948 - | 0.983 |
| 240 | 0.651 | 0.512 - | 0.784 | 0.969 | 0.949 - | 0.984 |
| 260 | 0.651 | 0.512 - | 0.784 | 0.969 | 0.949 - | 0.984 |
| 280 | 0.648 | 0.512 - | 0.78 | 0.971 | 0.952 - | 0.985 |
| 300 | 0.621 | 0.486 - | 0.763 | 0.971 | 0.954 - | 0.985 |
| 320 | 0.563 | 0.433 - | 0.706 | 0.972 | 0.955 - | 0.987 |
| 340 | 0.55 | 0.421 - | 0.694 | 0.975 | 0.958 - | 0.989 |
| 360 | 0.55 | 0.421 - | 0.694 | 0.975 | 0.958 - | 0.989 |
| 380 | 0.545 | 0.419 - | 0.685 | 0.98 | 0.966 - | 0.99 |
| 400 | 0.548 | 0.421 - | 0.693 | 0.981 | 0.967 - | 0.992 |
| 450 | 0.489 | 0.368 - | 0.624 | 0.982 | 0.969 - | 0.994 |
| 500 | 0.461 | 0.344 - | 0.596 | 0.98 | 0.967 - | 0.992 |

Table S9. DPP serum estimates of sensitivity and specificity with test median and 95% credible interval from posterior distributions a Bayesian model. Estimates are over a range of possible cut-off values at which to determine if a test is positive.

| **Cut-off (RLU)** | **Sensitivity** | | | **Specificity** | | |
| --- | --- | --- | --- | --- | --- | --- |
|  | **Median** | **95% CI** | | **Median** | **95% CI** | |
| 10 | 0.697 | 0.598 - | 0.794 | 0.927 | 0.905 - | 0.946 |
| 20 | 0.619 | 0.52 - | 0.724 | 0.947 | 0.927 - | 0.965 |
| 30 | 0.597 | 0.494 - | 0.706 | 0.956 | 0.939 - | 0.971 |
| 40 | 0.556 | 0.453 - | 0.669 | 0.962 | 0.945 - | 0.977 |
| 50 | 0.518 | 0.419 - | 0.626 | 0.966 | 0.95 - | 0.98 |
| 60 | 0.515 | 0.416 - | 0.623 | 0.968 | 0.952 - | 0.982 |
| 70 | 0.517 | 0.419 - | 0.623 | 0.971 | 0.955 - | 0.985 |
| 80 | 0.501 | 0.402 - | 0.6 | 0.975 | 0.959 - | 0.988 |
| 90 | 0.477 | 0.384 - | 0.584 | 0.975 | 0.96 - | 0.989 |
| 100 | 0.479 | 0.383 - | 0.586 | 0.975 | 0.961 - | 0.988 |
| 120 | 0.446 | 0.353 - | 0.551 | 0.977 | 0.963 - | 0.989 |
| 140 | 0.439 | 0.346 - | 0.541 | 0.978 | 0.964 - | 0.99 |
| 160 | 0.425 | 0.334 - | 0.524 | 0.98 | 0.966 - | 0.992 |
| 180 | 0.414 | 0.325 - | 0.515 | 0.98 | 0.966 - | 0.992 |
| 200 | 0.387 | 0.304 - | 0.486 | 0.979 | 0.967 - | 0.992 |
| 220 | 0.387 | 0.304 - | 0.486 | 0.979 | 0.967 - | 0.992 |
| 240 | 0.387 | 0.304 - | 0.486 | 0.979 | 0.967 - | 0.992 |
| 260 | 0.387 | 0.304 - | 0.486 | 0.979 | 0.967 - | 0.992 |
| 280 | 0.377 | 0.295 - | 0.478 | 0.98 | 0.966 - | 0.992 |
| 300 | 0.383 | 0.295 - | 0.479 | 0.982 | 0.969 - | 0.995 |
| 350 | 0.375 | 0.291 - | 0.469 | 0.984 | 0.97 - | 0.997 |
| 400 | 0.377 | 0.293 - | 0.474 | 0.985 | 0.97 - | 0.997 |
| 450 | 0.371 | 0.287 - | 0.472 | 0.986 | 0.973 - | 0.999 |
| 500 | 0.371 | 0.287 - | 0.472 | 0.986 | 0.973 - | 0.999 |


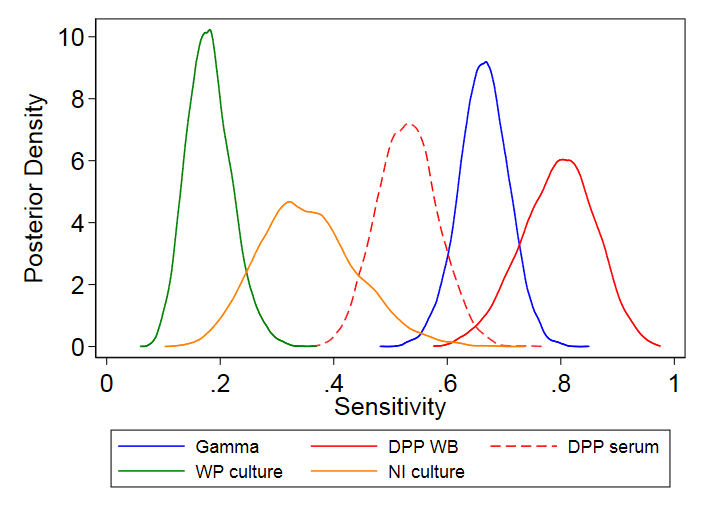


Figure S1. Posterior densities for diagnostic test sensitivity for various tests to detect *M. bovis* infection in live badgers, from the result of applying a Bayesian model to study data without assuming a gold standard.


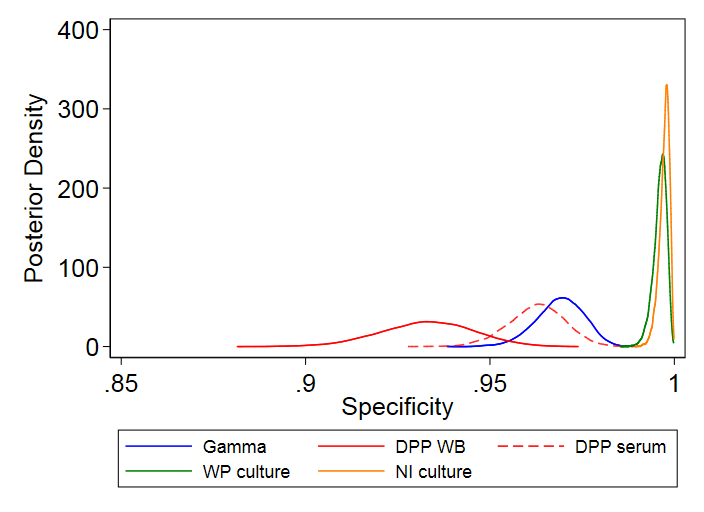


Figure S2. Posterior densities for diagnostic test specificity for various tests to detect *M. bovis* infection in live badgers, from the result of applying a Bayesian model to study data without assuming a gold standard.
